# Presbyvestibulopathy, Comorbidities, and Perception of Disability: A Cross-Sectional Study

**DOI:** 10.3389/fneur.2020.582038

**Published:** 2020-10-30

**Authors:** Andrés Soto-Varela, Marcos Rossi-Izquierdo, María del-Río-Valeiras, Isabel Vaamonde-Sánchez-Andrade, Ana Faraldo-García, Antonio Lirola-Delgado, Sofía Santos-Pérez

**Affiliations:** ^1^Division of Neurotology, Department of Otorhinolaryngology, Complexo Hospitalario Universitario, Santiago de Compostela, Spain; ^2^Department of Surgery and Medical-Surgical Specialities, University of Santiago de Compostela, Santiago de Compostela, Spain; ^3^Department of Otorhinolaryngology, University Hospital Lucus Augusti, Lugo, Spain; ^4^Department of Otorhinolaryngology, Complexo Hospitalario Universitario, Santiago de Compostela, Spain

**Keywords:** presbyvestibulopathy, comorbidities, disability, handicap, dizziness handicap inventory, DHI

## Abstract

**Objective:** To assess the perception of disability in patients with presbyvestibulopathy and to determine the factors (demographic, balance test scores, and comorbidities) that determine higher levels of disability.

**Material and Methods:** This was a cross-sectional study conducted in a tertiary university hospital. There were 103 patients who fulfilled the diagnostic criteria for presbyvestibulopathy and were included. Dizziness Handicap Inventory (DHI) score was the main variable used to quantify disability. Influence on DHI score, sex, age, time of evolution, equilibriometric parameters (posturographic scores and timed up and go test), history of falls, comorbidities (high blood pressure, diabetes, and dyslipidemia), psychotropic drug use, tobacco or alcohol use, living environment (urban or rural), and active lifestyle were analyzed.

**Results:** Most of the DHI scores showed a moderate (46 patients, 44.7%) or severe (39 participants, 37.9%) handicap. DHI scores were higher in women (59.8 vs. 36.1, *p* < 0.001), patients with obesity (58.92 vs. 48.68; *p* = 0.019), benzodiazepine (59.9 vs. 49.1, *p* = 0.008) or other psychotropic drug (60.7 vs. 49.2, *p* = 0.017) users, and fallers (57.1 vs. 47.3, *p* = 0.048). There was also a significant positive correlation between DHI score, time (Rho coefficient: 0.371, *p* < 0.001), and steps (Rho coefficient: 0.284, *p* = 0.004) used in the TUG and with the short FES-I questionnaire (a shortened version of the Falls Efficacy Scale-International) score (Rho coefficient: 0.695, *p* < 0.001). DHI scores were lower in alcohol consumers than in non-drinkers (46.6 vs. 56, *p* = 0.048). No significant correlation was found between DHI scores and age, time of evolution, posturographic scores, comorbidities, environment (rural or urban), or active lifestyle.

**Conclusion:** Most patients with presbyvestibulopathy show an important subjective perception of disability in relation to their symptoms. This perception is substantially higher in women than in men. The most influential factors are difficulties in walking, fear of falling, and obesity.

**Unique Identifier:** NCT03034655, www.clinicaltrials.gov.

## Introduction

Vestibular symptoms in the elderly are common and may result in reduced quality of life of these individuals (1). In addition, their consequences (primarily limiting mobility and increasing the risk of falls) are especially serious in this age group, leading to social isolation, and to direct morbidity and mortality (derived from eventual fractures caused by falls) (2). Health care for these patients is a major challenge for public healthcare systems (3).

The causes of vestibular symptoms in the elderly are varied (4, 5). Some highly prevalent clinical disorders (such as benign paroxysmal positional vertigo, BPPV) are more common in the elderly than in younger adults. Exposure to drugs or other vestibulotoxic substances is more likely and has a cumulative effect on people who are older because aging increases the likelihood of exposure to these substances. Various medications frequently used in older people (such as benzodiazepines and other central nervous system depressants) may slow vestibular reflexes. The existence of diseases that affect other systems (visual, locomotor, neurological, and cardiovascular, among others) may give rise to symptoms of dizziness or instability, which trigger (or potentiate) strictly vestibular symptoms (6). Lastly, aging itself may cause histologically demonstrable damage to vestibular receptors and organs (7–9) which may be responsible for the symptoms of dizziness, imbalance, or instability frequently reported by elderly people.

The physiological deterioration associated with aging has been referred to by various names in recent decades (including presbystasis, presbyvertigo, presbyequilibrium, and geriatric dizziness) (2). However, until 2019, there was no consensus on its description, and its existence was not generally accepted. The publication of the diagnostic criteria for presbyvestibulopathy by the Bárány Society in that year (10) has enabled an adequate characterization and homogenization of these patients, which has facilitated their study. Presbyvestibulopathy is almost invariably associated with other functional disorders in these patients, due to either aging itself (such as presbyopia or presbycusis) or the coexistence of other diseases (locomotor, neurological, and cardiovascular, among others). Therefore, it is not easy to assess how much of the disability that elderly people perceive regarding vestibular symptoms directly results from the aging of this system and how much of it results from other superimposed factors (comorbidities, use of central nervous system depressant drugs, the living environment of the patients and their level of physical activity, among others).

Different instruments can be used to measure disability caused by vestibular symptoms. The most commonly used instrument is the Dizziness Handicap Inventory (DHI) (11), developed in 1990 by Jacobson and Newman. Validated in different languages (including Spanish) (12), the DHI includes 25 questions divided into three groups (9 referred to the functional scale, 9 to the emotional scale, and 7 to the physical scale). Each of these 25 questions has three possible answers, which are scored as follows: “yes” (4 points), “sometimes” (2 points), and “no” (0 points). A score of 100 would indicate an absolute perception of disability, whereas a score of 0 would indicate that the subject does not perceive any disability. Total scores lower than 30 indicate mild disability, from 31 to 60 moderate disability, and higher than 60 severe disability (13).

The objective of this study was to evaluate the perception of disability in a sample of patients with presbyvestibulopathy who live in the community and to identify its determining factors (demographic, balance, comorbidities, and drugs, among others).

## Materials and Methods

This study was part of a clinical trial funded by the project PI1500329, integrated into the Spanish State Plan for R + D + I and funded by the Instituto de Investigación en Salud Carlos III- ISCIII -Subdirección general de Evaluación y Fomento de la Investigación and the Fondo Europeo de Desarrollo regional (FEDER). This clinical trial, the full protocol for which has already been published (14), aims to determine whether vestibular rehabilitation is useful in elderly patients with instability for improving their balance and reducing their risk of falling.

### Study Design

This was an observational cross-sectional study, conducted at the Otoneurology Unit of a tertiary hospital.

### Study Population: Inclusion and Exclusion Criteria

The total sample of the previously mentioned clinical trial was the initial population, consisting of individuals older than 65 years, with postural instability, who lived in the community (not institutionalized) and who met at least two of the following inclusion criteria:

(a) Having suffered at least one fall in the last 12 months.
(b) Taking more than 15 s or requiring a walking aid to complete the “timed up and go” test (15) (specific normality threshold calculated in previous studies).
(c) Having a mean balance percentage in the sensory organization test (SOT) of dynamic posturography (PD) < 68%.
(d) Having suffered at least one fall in the SOT of PD.
(e) Scoring 60% or higher in the Vertiguard geriatric Standard Balance Deficit Test (gSBDT).

The following exclusion criteria were used:

(a) Cognitive impairment or reduced cultural level, which prevented the patient from understanding the examinations and from giving their informed consent. All patients underwent a medical history, including questions about their symptoms. Specifically, those who were found to have difficulties in understanding the DHI items were excluded.
(b) Organic diseases, which prevented standing, which was necessary for balance assessment.
(c) Balance disorders caused exclusively by diseases other than age (neurological and vestibular, among others).

In all cases, imbalance was the symptom for which they consulted. A part of the patients was referred from Primary Care to the Otoneurology Unit; the rest were referred from the Department of Neurology. From this initial population (180 patients), the subjects who met the following diagnostic criteria of presbyvestibulopathy (10) were selected:

A. Chronic vestibular syndrome (at least 3 months duration) with at least 2 of the following symptoms:
  1. Postural imbalance or unsteadiness
  2. Gait disturbance
  3. Chronic dizziness
  4. Recurrent falls.
B. Mild bilateral peripheral vestibular hypofunction documented by at least 1 of the following:
  1. VOR gain measured by video-HIT between 0.6 and 0.8 bilaterally
  2. VOR gain between 0.1 and 0.3 upon sinusoidal stimulation on a rotatory chair (0.1 Hz, Vmax = 50–60°/s)
  3. Reduced caloric response (sum of bithermal maximum peak SPV on each side between 6 and 25°/s).
C. Age ≥60 years
D. Not better accounted for by another disease or Disorder

In total, 103 individuals met these criteria, forming the study population.

### Sample Size Estimation

To assess whether the sample size was sufficient to draw statistically significant conclusions, an estimate was performed, using the mean DHI score as a reference. From a previous study in our research group (16), the estimated standard deviation for this value was 18. A difference in score between study groups of 10 points was considered relevant. With a 95% confidence level (1-$\overset{´}{\alpha }$) and a 0.5 probability of type II error (β), for a bilateral hypothesis test, 84 subjects were deemed necessary. Therefore, the available sample (103 individuals) was considered sufficient to establish the existence of significant differences.

### Samples

The sample consisted of 103 patients with postural instability, who met the inclusion criteria. The mean age was 78.19 ± 5.72 years, with a minimum of 65.17 and a maximum of 92.31 years. Of the study population 77 patients were women (74.8%) and 26 were men (25.2%); the female/male ratio was 2.96/1.

### Method

A clinical history and a complete vestibular evaluation were performed to detect the causes of vestibular symptoms different from aging; This evaluation also served to confirm that the patients met the diagnostic criteria for presbyvestibulopathy. The examination included:

- Detection (or absence) of spontaneous nystagmus using Frenzel glasses: its presence was an exclusion criterion.
- Positional tests to detect benign paroxysmal positional vertigo (its presence did not necessarily mean the exclusion of the patient from the study; the patient was included in the study protocol when, once BPPV had resolved, symptoms and exploratory data consistent with presbyvestibulopathy persisted).
- Evaluation of the vestibulo-ocular reflex, through clinical (mainly, the cephalic impulse test) and instrumental (video Head Impulse Test and/or bithermal caloric tests) tests:
  ◦ video Head Impulse Test (vHIT): A portable video-oculography system (vHIT, GN Otometrics, Denmark), a high-speed infrared camera (250 Hz), and an accelerometer were used to measure movements of the right eye and head, during the cephalic impulses, in the horizontal plane. The head speed ranged from 150 to 240°/s, with amplitudes ranging from 15 to 20° from the center to the lateral position. Twenty records were collected and processed on each side, evaluating gain and the presence or absence of refixation saccades.
  ◦ Bithermal caloric testing (videonystagmograph HIS model, France) using water, with the following sequence: irrigation of left ear at 44°C, irrigation of right ear at 44°C, irrigation of left ear at 30°C, and irrigation of right ear at 30°C. The reflectance of each of the two ears was evaluated (sum of the mean speed of the slow phase of the nystagmus, at the maximum peak response, of the two stimulations of each ear).
- All the patients underwent a clinical neurological examination. When necessary (due to suspicion of neurological disease that could cause relevant symptoms), imaging (brain magnetic resonance imaging) was performed.

To evaluate balance (necessary to determine whether the patients met the inclusion criteria of the initial research project, from which the sample of the present study was obtained), the following tests were performed:

(a) A modified version of the Timed Up and Go (mTUG) test (15): the patient, sitting in a chair, must stand up without using their hand to push up, walk 3 m, turn around, walk around the chair, and sit down again. The time spent and the number of steps necessary to complete the test are quantified.
(b) The computerized dynamic posturography—sensory organization test (CDP-SOT) (Neurocom Smart Equitest platform): The SOT included quantitation of the patient's center of gravity (COG) displacements in 6 different sensorial information conditions as follows:
  - 1: fixed surface and visual surround, with eyes open.
  - 2: fixed surface, with eyes closed.
  - 3: fixed surface and moving visual surround, with eyes open.
  - 4: moving surface and fixed visual surround, with eyes open.
  - 5: moving surface, with eyes closed.
  - 6: moving surface and visual surround, with eyes open. Each of the six conditions was repeated three consecutive times, with the participants completing a total of 18 trials. The time established for each of these trials was 20 s.
(c) Limits of stability in the CDP (CDP-LOS): Following visual feedback (movement of a pictogram representing the subject's COP on a TV monitor), the patient had to voluntarily move his or her COP without moving his or her feet on the platform, to reach eight points around him/her. These points represented 100% of the displacement limit of the subject's COP, according to height and age.
(d) Balance record study using the mobile Vertiguard system (Vesticure GmbH, Germany): The following 14 tests were performed, and the analysis of the results represented the gSBDT:
  - Standing still (SS), with eyes open, on a normal surface (NS).
  - SS, with eyes closed, on a NS.
  - SS, one leg, eyes open, NS
  - Making 8 steps in tandem, with eyes open, on a NS.
  - SS, with eyes open, on a foam surface (FS).
  - SS, with eyes closed, on a FS.
  - Making 8 steps in tandem, with eyes open, on a FS.
  - Walking 3 m, with eyes open.
  - Walking 3 m, with eyes open, while turning the head from side to side.
  - Walking 3 m, with eyes open, while moving the head up and down.
  - Walking 3 m, with eyes closed.
  - Walking over 4 barriers (height: 26 cm; distance between barriers: 1 m).
  - Sitting down on a chair.
  - Getting up from a chair.
(e) A questionnaire assessing the perception of disability in relation to postural instability: the Dizziness Handicap Inventory (DHI), validated in Spanish (12), and previously explained in the introduction of this manuscript.
(f) A questionnaire assessing the fear of falling: a shortened version of the Falls Efficacy Scale-International to assess fear of falling (Short FES-I) (17). It evaluates fear of falling while performing 7 everyday activities. Each question has 4 possible answers scored as follows: “not at all concerned” (0 points), “somewhat concerned” (1 point), “quite concerned” (2 points), and “very concerned” (3 points). The highest score (greatest fear of falling) is 21, and the lowest is 0.
(g) The patients were directly asked whether they had suffered a fall in the previous 12 months and, if the answer was yes, the number of falls during this time.

The balance tests (mTUG, CDP, and Vertiguard) were carried out by trained personnel in vestibular assessment. In all of them, before performing the test, the patient received a detailed explanation and an initial training record was made. Next, the tests were carried out according to the protocol followed in our clinic: three trials in each task for the CDP-SOT and one each for the mTUG, CDP-LOS, and gSBDT Vertiguard. The questionnaires were delivered in writing to the patient (after an explanation by the researcher), who answered them on their own or with the help of a family member.

### Study Variables

The main variable was the DHI score, considered a continuous (score) and discontinuous (mild disability, 30 points or lower; moderate disability, from 31 to 60 points; and severe disability, 60 points or higher) variable (13). The scores of each DHI scale (functional, emotional, and physical) were considered secondary variables.

The relationships of the DHI score with the following variables were analyzed:

a) Sex.
b) Age.
c) Age at onset of symptoms.
d) Time of symptom progression (in months).
e) Body mass index: weight (in kg)/ height^2^ (in meters), according to which the participants were divided into obese (BMI ≥ 30) and non-obese (BMI < 30).
f) Falls in the previous 12 months: number of falls, dividing patients into fallers (at least one fall) vs. non-fallers (no falls), and also dividing them into recurrent fallers (more than one fall) vs. non-recurrent fallers (no or one fall) (10).
g) mTUG: time and steps necessary to complete the test.
h) CDP-SOT:
  - The equilibrium score for each condition (the arithmetic mean of the three entries for each condition).
  - The composite equilibrium score, which was calculated as the weighted average of the 18 SOT scores.
  - The effectiveness of somatosensory input use, which was a percentage value from the application of the following formula: (average score of condition 2/average score of condition 1) × 100.
  - The effectiveness of visual input use, which was calculated using the following formula: (average score of condition 4/average score of condition 1) × 100.
  - The effectiveness of vestibular input use, which was assessed using the following calculation: (average score of condition 5/average score of condition 1) × 100.
  - The ability to assume erroneous visual input, a score was assigned using the following calculation made using the values determined by the conditions: [(2+5)/(3+6)] × 100.
i) CDP-LOS:
  - Maximum excursion (ME): measurement of the maximum COP excursion, with respect to 100% of the theoretical limit of stability (as a percentage).
  - Endpoint excursion (EE): measure of the distance achieved toward a target on the initial movement (as a percentage).
  - Directional control: comparison between movement in the direction of the target vs. movement away from that direction, as a percentage. A value of 100% would be a straight line from COP to the intended target.
j) Short FES-I score.
k) Association with BPPV (with diagnosis confirmed by positional tests).
l) Association with comorbidities (detected by directly asking patients and by consulting their electronic medical records):
  - Obesity (calculated according to the BMI).
  - Heart disease (primarily hypertensive and/or ischemic heart disease).
  - Diabetes mellitus.
  - Neurological disease (primarily ischemia or Parkinson's disease).
m) Use of psychotropic drugs (benzodiazepines and other psychotropic drugs), by directly asking patients and by consulting their electronic medical records.
n) Consumption of alcohol, by directly asking the patients.
o) Development of an active (with the ability to walk without assistance and to be independent to perform the basic activities of daily life) or inactive (participants who need help walking and/or performing basic activities of daily life) lifestyle.
p) Living environment (rural vs. urban). The environment was defined according to legal and administrative criteria. In Spain, a rural environment is defined as that whose population is <5,000 inhabitants. The demographic data of each population were retrieved from the population records available in the National Institute of Statistics database (Instituto Nacional de Estadística—INE).

### Statistical Analysis

Fisher's exact test was used to analyze the relationship between nominal variables, in 2 × 2 contingency tables, calculating the odds ratio and 95% confidence intervals. The Chi-squared test was used to analyze the relationship between nominal variables and the DHI score (as a categorical variable). The Kolmogorov-Smirnov test was used to determine whether the continuous variables followed a normal distribution. When this test confirmed the hypothesis of normality, a Student's *t*-test was used to relate them to the nominal variables. Conversely, when the continuous variables did not follow a normal distribution, a non-parametric Mann-Whitney *U*-test was used to examine these relationships. To assess the effect of the variables sex (males and females), associated neurological disease, Benzodiazepine use, use of other psychotropic drugs, and alcohol consumption (yes vs. no) on DHI, generalized linear models (GLM) were used. The Akaike Information Criteria (AIC) corrected for finite samples was used as goodness-of-fit test of the models, and the Wald test was used to compare model effects. Finally, to correlate continuous variables with each other, Spearman's Rho correlation test was used. The level of statistical significance for all tests was set at *p* < 0.05.

The SPSS 15.0 software for Windows was used for the statistical analyses.

### Ethical Aspects

The protocol was approved by the Independent Ethics Committee of Galicia (protocol No. 2014/411). The study was conducted in accordance with the ICH Good Clinical Practices, the Declaration of Helsinki, and Law 14/2007 of 3 July on Biomedical Research. All the patients signed a written informed consent form to participate in the study.

## Results

The mean DHI score of the sample was 53.65 ± 22.28. The mean score on the functional scale was 22.37 ± 9.35, the emotional scale was 14.78 ± 8.52, and the physical scale was 16.50 ± 7.71. With respect to the total DHI score, 18 patients (17.5%) had a perception of mild disability, 46 (44.7%) of moderate disability, and 39 (37.9%) of severe disability.

Regarding the demographic and clinical variables, the mean BMI was 29.88 kg/m^2^ ± 4.24, the mean age at onset of symptoms was 75.03 ± 6.16 years, and the mean time of symptom progression was 3.17 ± 3.15 years. The mean number of falls suffered in the previous 12 months was 8.36 ± 36.78. The distribution of the other variables is outlined in Table 1.

**Table 1:** Distribution of history of falls, comorbidities, psychotropic drug use, tobacco or alcohol use, living environment, and active lifestyle.

**Variable**	**Yes**	**No**
Faller	70 (67.96%)	33 (32.04%)
Recurrent faller	51 (49.51%)	52 (50.49%)
BPPV	5 (4.85%)	98 (95.15%)
Heart disease (hypertensive and/or ischemic)	66 (64.08%)	37 (35.92%)
Diabetes mellitus	25 (24.27%)	78 (75.73%)
Dyslipidemia	58 (56.31%)	45 (43.69%)
Associated neurological disease	17 (16.50%)	86 (83.50%)
Benzodiazepine use	46 (44.66%)	57 (55.34%)
Consumption of other psychotropic drugs	43 (41.75%)	60 (58.25%)
Tobacco use	4 (3.88%)	99 (96.12%)
Alcohol consumption	23 (22.33)	80 (77.67%)
Active lifestyle	98 (95.15%)	5 (4.85%)
Rural environment	68 (66.02%)	35 (33.98%)

The DHI score was affected by the sex of the patients (higher mean score in women than in men: 59.77 vs. 36.08; *p* = 4.06 e^−7^, Student's *t*-test). Conversely, no correlation of the DHI score with the age of the patient (*p* = 0.824, coefficient value = −0.022), age of symptom onset (*p* = 0.596, coefficient value = −0.053) or the time of progression of these symptoms (*p* = 0.348, coefficient value = 0.093) was detected by Spearman's Rho correlation.

Correlations were detected between the DHI score and the number of falls suffered in the last year (*p* = 0.009, coefficient value = 0.255; Spearman's Rho correlation), between being a faller or non-faller (higher mean DHI score in fallers than in non-fallers: 57.13 vs. 47.33; *p* = 0.048, Student's *t*-test), and between being a recurrent or non-recurrent faller (higher mean score in recurrent-fallers: 59.18 vs. 48.23, *p* = 0.012, Student's *t*-test).

Regarding the balance evaluation, a Spearman's Rho correlation showed no relationship between the DHI score and most measures of dynamic posturography. As shown in Table 2, only the score of condition 2 of the sensory organization test showed some correlation with the DHI score. In turn, the TUG values were significantly correlated with both the time (*p* = 0.0001, coefficient value = 0.371) and the number of steps necessary to complete the test (*p* = 0.004, coefficient value = 0.284). Fear of falling, measured using the short FES-I questionnaire, was strongly correlated with the DHI score (*p* = 3.95 e^−16^, coefficient value = 0.695).

**Table 2:** Correlation between DHI score and most relevant CDP scores (Spearman's Rho correlation).

	**Parameter**	**Coefficient value**	***P*-value**
CDP sensory organization test	Overall average balance	−0.142	0.152
	Condition 2	−0.218	0.027
	Condition 5	−0.031	0.754
	Somatosensory input	−0.131	0.186
	Visual input	−0.153	0.122
	Vestibular input	−0.026	0.795
	Visual preference	0.003	0.976
CDP limits of stability	Endpoint excursion	−0.083	0.404
	Maximum excursion	−0.073	0.461
	Directional control	−0.085	0.392

No relationship was detected between the DHI score and any of the following study variables: presence of BPPV (*p* = 0.381, Mann–Whitney *U*-test), hypertensive and/or ischemic heart disease (*p* = 0.791, Student's *t*-test), diabetes mellitus (*p* = 0.798, Student's *t*-test), practicing physical activity (*p* = 0.275, Student's *t*-test), or living in a rural or urban environment (*p* = 0.142, Student's *t*-test).

Higher DHI scores were related to obesity (higher mean score in obese patients, 58.92 vs. 48.68; *p* = 0.019, Student's *t*-test), absence of associated neurological disorders (higher mean score in those without associated neurological disorders than in those with such disorders, 55.84 vs. 43.88; *p* = 0.016, Student's *t*-test), benzodiazepine use (49.07 mean in non-users vs. 59.91 in users; *p* = 0.008, Student's *t*-test), use of other psychotropic drugs (49.23 mean score in non-users vs. 60.71 in users; *p* = 0.009, Mann–Whitney *U*-test) and absence of alcohol consumption (46.64 mean score in alcohol drinkers vs. 55.98 in teetotalers; *p* = 0.048, Student's *t*-test). Some of these variables were affected by sex. Associated neurological disorders were more frequent in men (34.6%) than in women (10.4%), with *p* = 0.007 [Fisher's exact test; OR = 4.566, 95% CI (1.535; 13.585)]. Benzodiazepine use was much less frequent in men than in women [19.2 vs. 53.2%; *p* = 0.002, Fisher's exact test; OR = 0.209, 95% CI (0.071; 0.611)]. Lastly, 53.8% of the men consumed alcohol often, whereas only 11.7% of the women do so [*p* = 3.24 e^−5^, Fisher's exact test; OR = 8.815, 95% CI (3.121; 24.894)].

Multivariate analysis including sex, obesity, absence of neurological disease and benzodiazepine use, use of other psychotropic drugs, and alcohol consumption (Table 3 presents the final generalized linear model) shows that sex, followed by obesity, was the variable that most significantly associated with an increase in DHI score. Female sex was associated with an increase in DHI by 20.29 points (95% CI 10.27; 30.32) compared to the male sex, whereas being obese increased the DHI score by 8.47 points (95% CI 1.31; 15.629) in comparison with those who were not obese.

**Table 3:** Multivariate analysis evaluating how sex, obesity, absence of neurological disease and benzodiazepine use, use of other psychotropic drugs, and alcohol consumption, influence DHI score.

**Variable**	**Coefficient (95% CI)**	**Wald's Chi square**	***P*-value**
Sex (female)	19.273 (9.459; 29.086)	14.816	0.0001
Associated neurological disease (no)	7.035 (−3.110; 17.179)	1.847	0.174
Benzodiazepine use (no)	−4.719 (−12.242; 2.804)	1.511	0.219
Consumption of other psychotropic drugs (no)	−7.424 (−15.910; −0.062)	3.778	0.052
Alcohol consumption (no)	−1.264 (−10.862; 8.335)	0.067	0.796
Obesity	8.468 (1.308; 15.629)	5.373	0.020
Intersection	37.176 (23.514; 50.838)	28.443	9.65 e^−8^

Lastly, the relationships between the scores of the three DHI scales (functional, emotional, and physical) and the variables that affected the total DHI score were analyzed. As shown in Table 4, the emotional scale was the most strongly affected by the study variables because its relationships were significant with all of them.

**Table 4:** Relationships between the scores of the three DHI scales (functional, emotional, and physical) and the variables that affected the total DHI score.

**Variables**	**Scales**		
	**Functional**	**Emotional**	**Physical**
Sex	***p*** **=** **3.49 e**^**−5**^ (Mann–Whitney *U*- test)	***p=8.95 e**^**−6**^* (Student's *t*-test)	***p=0.0004*** (Mann–Whitney *U*-test)
Number of falls (previous year)	*p* = 0.144, coefficient value = 0.145 (Spearman's Rho correlation)	***p=0.004, coefficient value=0.283*** (Spearman's Rho correlation)	***p=0.016, coefficient value=0.237*** (Spearman's Rho correlation)
Faller vs. non-faller	*p* = 0.519 (Mann–Whitney *U*-test)	***p=0.008*** (Student's *t*-test)	*P* = 0.304 (Mann–Whitney *U*-test)
Recurrent faller vs. no recurrent faller	*P* = 0.113 (Student's *t*-test)	***p=0.010*** (Student's *t*-test)	***p=0.044*** (Mann–Whitney *U*-test)
TUG (time)	***p=8.48 e**^**−5**^**coefficient value=0.377*** (Spearman's Rho correlation)	***p=0.005, coefficient value=0.272*** (Spearman's Rho correlation)	***p=0.002, coefficient value=0.300*** (Spearman's Rho correlation)
TUG (steps)	***p=0.002 coefficient value=0.296*** (Spearman's Rho correlation)	***p=0.022, coefficient value=0.225*** (Spearman's Rho correlation)	***p=0.045, coefficient value=0.198*** (Spearman's Rho correlation)
Short FES-I score	***p=1.12 e**^**−12**^**coefficient value=0.629*** (Spearman's Rho correlation)	***p=4.70 e**^**−13**^**, coefficient value=0.637*** (Spearman's Rho correlation)	***p=4.86 e**^**−9**^**, coefficient value=0.537*** (Spearman's Rho correlation)
Neurological disease	*P* = 0.055 (Mann–Whitney *U*-test)	***p=0.010*** (Student's *t*-test)	*p* = 0.059 (Mann–Whitney *U*-test)
Benzodiazepines	*P* = 0.072 (Mann–Whitney *U*-test)	***p=0.002*** (Mann–Whitney *U*-test)	***p=0.032*** (Mann–Whitney *U*-test)
Psychotropic drugs	***p=0.017*** (Student's *t*-test)	***p=0.006*** (Student's *t*-test)	*p* = 0.317 (Student's *t*-test)
Alcohol consumption	***p=0.041*** (Student's *t*-test)	***p=0.013*** (Student's t-test)	*p* = 0.753 (Mann–Whitney *U*-test)

*The bold values are statitistically significant*.

## Discussion

Symptoms related to aging of the vestibular system (presbyvestibulopathy) significantly limit the quality of life of the elderly due to their associated discomfort, reduced mobility, isolation, and morbidity (1). They are typically associated with aging related alterations in other sensory systems, such as presbyopia or presbycusis, which have been associated with an increased risk of falls, depression, and mortality (18–20). However, in elderly people, presbyvestibulopathy symptoms do not appear in isolation but typically occur in complex clinical and socio-family contexts. They are usually associated with comorbidities, which cause disability. Drugs that affect the central nervous system are frequently used at these ages, and their effect may be aggravated by the intake of toxic substances (such as alcohol). An individual's living environment may also influence the subjective perception of the disability that results from vestibular symptoms. Identifying the key elements that affect this disability, in the usually heterogenous clinical context of these patients, would make it possible to preferentially target them and improve the subjective perception of their own ability, their relationships with their family, and their social environment.

It is first important to realize that the perception of disability is high among patients with presbyvestibulopathy. Although not specifically referring to this condition, previous studies have already reported that elderly patients with dizziness present with decreased quality of life (21, 22). In our study, 82.6% of the individuals showed DHI scores that revealed moderate or severe disability. The limitation caused by presbyvestibulopathy is, therefore, important for the life of these patients, which further underscores the need to identify the factors that condition this disease and to appropriately target them. Moreover, the perception of disability is much higher in women than in men, an effect which is not affected by age. This may be explained by the differences that persist between the sexes in terms of work activities and responsibility for housework. In our society, men carry out their work essentially outside the home and take on less household chores. When they reach 65 years (the age around which retirement typically occurs), their physical activity decreases substantially. Therefore, they perceive a lower disability due to vestibular symptoms. Conversely, retired women usually continue to lead a much more active life (housework and taking care of grandchildren, among other activities). Thus, the same vestibular symptoms may imply a greater disability in their day-to-day life, compared to men. This gender difference in roles has decreased significantly in recent decades, but it is still evident in the elderly population today.

Regarding the factors that are most directly related to vestibular symptoms, falls show the clearest relationship with the perception of disability. The number of falls suffered in the previous 12 months, the fact of having fallen infrequently or, particularly, frequently, and the fear of falling (measured using the short FES-I questionnaire), are all parameters related to a higher DHI score. This relationship between falls and DHI has been previously reported in a group of elderly patients with postural instability (albeit without meeting the presbyvestibulopathy criteria and which had not yet been published at that time) (23). Therefore, all actions aimed at reducing (or avoiding) falls and the fear of falling may lead to a significant decrease in the perception of disability. They will also make it possible to break the vicious cycle of falling-fear and falling-disability-decreased mobility-increased risk of falling. Along the same lines, poor TUG scores (in both time and steps necessary to complete the test) are strongly correlated with DHI score. Therefore, walking difficulties increase the perception of disability and, as such, improving this mobility is essential to increase feelings of security.

However, the dynamic posturography results are unrelated to the perception of disability in patients with presbyvestibulopathy. Regarding this possible relationship, although not specifically referring to elderly patients, discordant results have been published in the literature. Some authors find no relationship between DHI and posturography (24), whereas others report low (25) or moderate (26) correlations. In elderly patients with postural instability (without meeting presbyvestibulopathy criteria, albeit not yet published at the time), a relationship was found between DHI and the scores of a mobile posturographic system (Sway Star), but not between DHI and computerized dynamic posturography (23). This lack of relationship detected in our sample may be due to the fact that, although dynamic posturography is a good method for assessing and quantifying balance, it does not assess stability when walking. Falls in elderly individuals (the key parameter related to DHI score) do not usually occur in static situations but when walking or moving body position, thereby accounting for this lack of correlation between posturography and perception of disability in the elderly with presbyvestibulopathy.

The relationship (or lack thereof) between the DHI score and most comorbidities analyzed in this study stands out. The DHI score is indeed related to obesity, with worse scores in individuals with presbyvestibulopathy and BMI >30. The relationship between obesity and worsening of balance in elderly people with postural instability has already been published (albeit without meeting presbyvestibulopathy diagnostic criteria, which had not yet been defined) (27). Moreover, the existence of another associated vestibular disease (in this case, BPPV), heart disease, dyslipidemia, or diabetes has no effect on the DHI score. Although this questionnaire aims to measure the perception of disability regarding vestibular symptoms, the accumulation of diseases should heighten the perception of disability, but this does not occur in our sample, perhaps because, when properly treated, these comorbidities do not necessarily imply an increased perception of disability. Surprisingly, the presence of associated neurological diseases (Parkinson's disease and history of ischemic heart disease, among other conditions) were associated with lower DHI scores (when the opposite was expected). This may be because the neurological damage masks presbyvestibulopathy symptoms, which go more unnoticed by the patient. As a result, patients may blame other conditions for the eventual disability.

The association between the use of benzodiazepines and other psychotropic drugs and worse DHI scores was expected. Beyond the underlying disease that determines the use of these drugs (which can increase the perception of disability), central nervous system depressant drugs slow vestibular reflexes, aggravating presbyvestibulopathy symptoms. Therefore, whenever possible, the use of these drugs should be limited in elderly patients with presbyvestibulopathy because they worsen the perception of their functional capacity. The lower scores in patients who consume alcohol may be due to their lower awareness of their limitations considering the effects of alcohol on the central nervous system. Tobacco use had no effect on DHI scores, which is not relevant because almost the entire sample (96%) consisted of non-smokers (likely due to the predominance of women, among whom smoking is less prevalent than among men).

In turn, living in a rural or urban environment had no effect on the perception of disability among these subjects. Considering their greater variety of sensory stimuli that may worsen the symptoms of presbyvestibulopathy, urban settings were expected to heighten the perception of disability, but we did not observe this difference in our sample. In fact, previous studies on elderly patients with postural instability (not necessarily due to presbyvestibulopathy) have also failed to find a relationship between DHI score and living in a rural or urban environment (28). The lack of effect of physical activity must be interpreted with great caution. In total, 95% of our patients maintained an active lifestyle (since our sample was chosen from a population of patients who were candidates for vestibular rehabilitation and who tolerated standing, which was one of the inclusion criteria). It would be interesting to analyze whether the perception of disability influences in any way the level of physical activity of the patients (in this study, we have divided them into active vs. inactive lifestyle, but the different degrees of physical activity have not been analyzed).

Questions regarding the emotional scale showed the highest score in relation to the study variables. Disability is, therefore, a problem more related to a subjective perception of limitation than to an actual physical and/or functional disability. This aspect must be considered in the therapeutic strategies that are designed to reduce this disability because emotional care for these patients could be essential in improving the perception of their own abilities.

The characteristics of our sample involve some limitations that force us to be cautious in generalizing the conclusions. All patients in our series were at least 65 years of age, whereas the diagnostic criteria for presbyvestibulopathy includes individuals 60 years and older. Subjects between 60 and 65 years of age are still of working age, so a relevant factor in their perception of disability may eventually be the effect of vestibular symptoms on their ability to work. In our sample, given the age of the patients, most patients were already retired, so this parameter was not analyzed in this study.

Another limitation refers to the quantification of falls in the previous 12 months. These data were collected by directly asking the patients and may therefore have a memory bias. The patients are likely to remember exactly whether they have fallen or not (fallers vs. non-fallers) and even whether they have fallen more than once (recurrent fallers vs. non-recurrent fallers), but it is more difficult to reliably pinpoint the exact number of falls.

A third limitation refers to the absence of an assessment for an anxiety disorder. It would have been interesting to have done it, especially after having detected the highest scores on the emotional subscale of the DHI. We have used benzodiazepine consumption (which is associated with higher DHI scores) as an indirect way of measuring anxiety, but a specific questionnaire quantifying it had been useful.

Finally, a fourth limitation refers to the absence of a systematic evaluation of hearing and vision in these patients. Most (not 100%) underwent pure tone audiometry; vision assessment was performed in a smaller percentage of patients. This is the reason why these variables, which have been related to the decrease in the quality of life of the elderly, have not been analyzed in this study.

Nonetheless, our results clearly show that most patients with presbyvestibulopathy (at least, those older than 65 years) perceive a moderate-to-severe disability regarding their vestibular symptoms and that the factors most strongly related to this perception are female sex, falls (the fact of having fallen, especially repeatedly, the number of falls, and the fear of falling), and mobility difficulties (measured using the TUG test), together with obesity.

In conclusion, we consider that an adequate weight control in these patients, as well as clinical intervention through vestibular rehabilitation programs for reducing falls (and the fear of suffering them) and improving mobility, will lead to a lower perception of disability and to an improved quality of life in elderly patients with presbyvestibulopathy.

## Data Availability Statement

The datasets generated for this study are available on request to the corresponding author.

## Ethics Statement

The studies involving human participants were reviewed and approved by Independent Ethics Committee of Galicia (protocol 2014/411). The patients/participants provided their written informed consent to participate in this study.

## Author Contributions

AS-V, MR-I, Md-R-V, AF-G, IV-S-A, Md-R-V, AL-D, and SS-P have contributed to the conception and design of this manuscript, revised it critically, approved the final version, and agree to be accountable for all aspects of the work in ensuring that questions related to the accuracy or integrity of any part of the work are appropriately investigated and resolved. AS-V has designed the protocol of the study. AF-G, IV-S-A, AL-D, and SS-P have performed the clinical and posturographic examination. Md-RV has collected and analyzed the data. MR-I has developed the statistical analysis. AS-V has written the manuscript. SS-P has revised critically the manuscript. All authors contributed to the article and approved the submitted version.

## Conflict of Interest

The authors declare that the research was conducted in the absence of any commercial or financial relationships that could be construed as a potential conflict of interest.
